# On the role of chance in fencing tournaments: An agent-based approach

**DOI:** 10.1371/journal.pone.0267541

**Published:** 2022-05-05

**Authors:** Chiara Zappalà, Alessandro Pluchino, Andrea Rapisarda, Alessio Emanuele Biondo, Pawel Sobkowicz

**Affiliations:** 1 Department of Physics and Astronomy, University of Catania and INFN Catania division, Catania, Italy; 2 Complexity Science Hub Vienna, Vienna, Austria; 3 Department of Economics and Business, University of Catania, Catania, Italy; 4 National Centre for Nuclear Research, Otwock, Poland; Institute of Molecular Genetics of Czech Academy of Sciences: Ustav molekularni genetiky Akademie Ved Ceske Republiky, CZECH REPUBLIC

## Abstract

It is a widespread belief that success is mainly due to innate qualities rather than external forces. This is particularly true in sports competitions, where individual talent is usually considered the main, if not the only, ingredient to reach success. In this study, we explore the limits of this belief by quantifying the relative weight of talent and chance in fencing, a combat sport involving a weapon, with the help of both real data and agent-based simulations. Fencing competitions are structured as direct elimination tournaments, where randomness is explicitly present in some rules. We focused on épée, which is one of three disciplines. We collected data on international competition results and annual rankings, in the range 2008–2020, for male and female fencers under 20 years old (Junior category). Then, we built the model calibrated on our dataset and parametrized by just one free variable *a*, describing the importance of talent—and, consequently, of chance—in competitions (*a* = 1 indicates the ideal scenario where only talent matters, *a* = 0 the complete random one). Our agent-based approach can reproduce the main stylized facts observed in data, at the level of both single tournaments and the entire careers of a given community of épée fencers. We find that simulations approximate very well the data for both Junior Men and Women when talent weights slightly less than chance, i.e. when *a* is around 0.45. We conclude that the role of chance in fencing is unusually high and it probably represents an extreme case for individual sports. Our findings shed light on the importance of external factors in both athletes’ results in tournaments and throughout their career, making even more unfair the “winner-takes-all” disparities that often occur between the winner and the other classified competitors.

## Introduction

Thinking about successful careers in general, and in sports in particular, the common belief is that they are just the result of hard work, endurance and effort combined with extraordinary skills. One could naively think they develop from a long series of successes in several competitions, after endless training sessions and great sacrifices, and that only predestined champions with innate uncommon abilities can get them. Inner talent, in this view, makes the main difference in career development.

However, many recent studies have shown that talent does not vary so widely among people and that its probability distribution is limited and concentrated around a well defined mean value [1–4].

Although individual talents do not differ that much from one person to another, only a few reach the top. We experience it in many fields [4–7] and clearly in sports [3, 8], where we observe how people suddenly get notoriety and often huge amounts of money when they win.

Even with comparable talents, usually very high, athletes could end up with totally different rewards in a competition. One event after another, small fortuitous differences might give rise to a cumulative advantage [9, 10], which generates a consistent increasing gap between individuals with similar talents. We tend to admire those who reach the top, disregarding others in the ranking. We are so used to this common “winner-takes-all” logic that it sounds inevitable, in sports, arts, even in science. And most often the rationale given for such selectivity is formulated in terms of innate talent, coupled with effort: “they are the best”.

We still lack a clear understanding of the processes which lead to vastly different rewards in sports and many other settings. In reality many small and unpredictable circumstances often play a role, yet as much often we tend to ignore those influences, preferring to believe in truly exceptional or gifted people. This talent-oriented view persists despite being rejected by a wealth of evidence [2–7, 11, 12].

In this paper, we investigate those effects in the context of an individual sport. In this kind of sports it is usually easier to analyse results and then assess the consequences, since many of them have simple rules and athletes act in a controlled environment. Thus, they are suitable for testing an agent-based approach which could reproduce, in a virtual setting, athletes’ performance and tournaments’ structure, capturing the role of individual abilities versus external circumstances in achieving success. We will specifically consider *fencing* as a case study, since it is an individual sport made up of face-to-face matches (called bouts) that directly compare athletes, underlining their similarities in contrast with huge differences in their outcome. Fencing is a combat sport involving a weapon, which can be of three different kinds, identifying three separate disciplines: épée, foil and sabre. We focus on épée because there is no right of way rule regarding attacks, which means that any hit (also called touch) is counted [13, 14].

Fencing is a perfect example of how unpredictable factors can strongly condition careers. First of all, randomness is explicitly present in its rules: in case of a tie, one extra minute is given and a priority is assigned at random to one fencer who could win the match “for free” if no valid touches are scored. Secondly, competitions are arranged as tournaments with two distinct phases (pools and direct elimination [see Fencing rules for details]), characterised by intensive bouts performed in short periods of time, with irregular breaks in between [15]. Thus, a competition can last an entire day and it is difficult to keep both physical and psychological energies under control; such a delicate aspect has been examined in several studies [15–20], highlighting the unique cognitive processes enhanced by fencing [21]. For our purposes, we point out that the organization of tournaments itself exposes fencers to the influence of random events, which may globally condition the day of the competition (e.g. sudden injuries, fever, irregular schedule of bouts) and/or simply affect the match outcome. Moreover, the rules for updating the seasonal ranking probably gives more importance to a single outstanding result, less regarding a constant and pretty decent career during the years.

To quantify all those effects, we introduce an agent-based model able to simulate young fencer career progression as function of a single control parameter *a*, which tunes the relative weight of talent and randomness in competitions. Then, we compare the numerical results with fencing data extracted from a real dataset, for both official rankings and single tournaments. We will show that, as expected, the role of chance in this sport discipline is significantly higher than commonly believed.

The paper is organized as follows: in Materials and methods we provide information on the adopted dataset, explain fencing rules and present our agent-based model for fencing tournaments; then, we show and analyse our findings in Results and Discussion; finally, we summarise our results and examine some of their implications in Conclusions. More details about our dataset and the calibration of the model itself can be found in S1 Appendix.

## Materials and methods

### Dataset

We collected the official Junior Men and Women rankings from 2011 to 2019, excluding previous years because there were very different criteria in point assignment and next years (2020–2021) since they are incomplete (many events have been cancelled due to COVID-19 pandemic). All participants are in between 14 and 20 years old and they are from all the countries that are members of the International Fencing Federation (FIE), whose website [13] stores those rankings we based our analysis on. If we look at the age limitation, the longest possible career in Junior category lasts six years. Since we have nine years available, the group of athletes involved is not constant over time. Instead, every season there are some fencers who become too “old” and must change their category; on the other hand, the “youngest” fencers who can participate in Junior Events for the first time come into play. For this reason, rankings do not have a fixed number of competitors; we find that the average length for Junior Men is about 600 participants, while for women is about 500 in our dataset (see S1 Appendix for details).

We also collected the initial rankings and final classifications of 100 World Cups, from 2008 to 2020, to perform a more detailed analysis of single tournaments (later shown in Results and Discussion). Data availability was a non trivial issue in this case, several sources were consulted to extend our dataset as much as possible [13, 22–28]. For this reason, we provide a repository that gather all the data we used [29].

### Fencing rules

As already mentioned in the Introduction, fencing is a combat sport that involves a weapon, which can be of three different kinds, identifying three separate disciplines: foil, sabre, épée (the source for this section is fie.org [13]).

Each weapon has its own peculiarities, but they also have common characteristics: the two opponents compete on a piste, 14 metres long and 1.5 metres wide; the goal is to score a valid touch on your opponent, which counts as a point; the first fencer that achieves 15 points, in a bout composed at most of three rounds (called periods) of three minutes each, wins; touches and time are controlled by a referee, according to an electrical recording apparatus.

In case of a tie at the end of the third period, one extra minute is given, randomly assigning a priority to one of the two fencers: if no one scores a single touch during this time, whoever has the priority wins. Therefore, the role of randomness is explicitly present in fencing rules, making this sport an interesting candidate in studying the effects of chance (good or bad luck, other external factors) in competitions.

In this work, we study épée because there are no right of way rules regarding attacks, which means that the point is always assigned to the fencer who makes the hit first. As a consequence, referee discretion is strongly limited and we can neglect human error contribution.

The main features of épée are the following:

- the attack is possible only with the point of the weapon;
- the target area is the entire body;
- since any touch is counted, double-hits (i.e. simultaneous touches) are allowed if they occur within 40 milliseconds.

Those very essential rules allow épée tournaments to be simulated by an agent-based model, as described in Model. But first, we need to explain the organization of fencing competitions.

**Fig 1:**
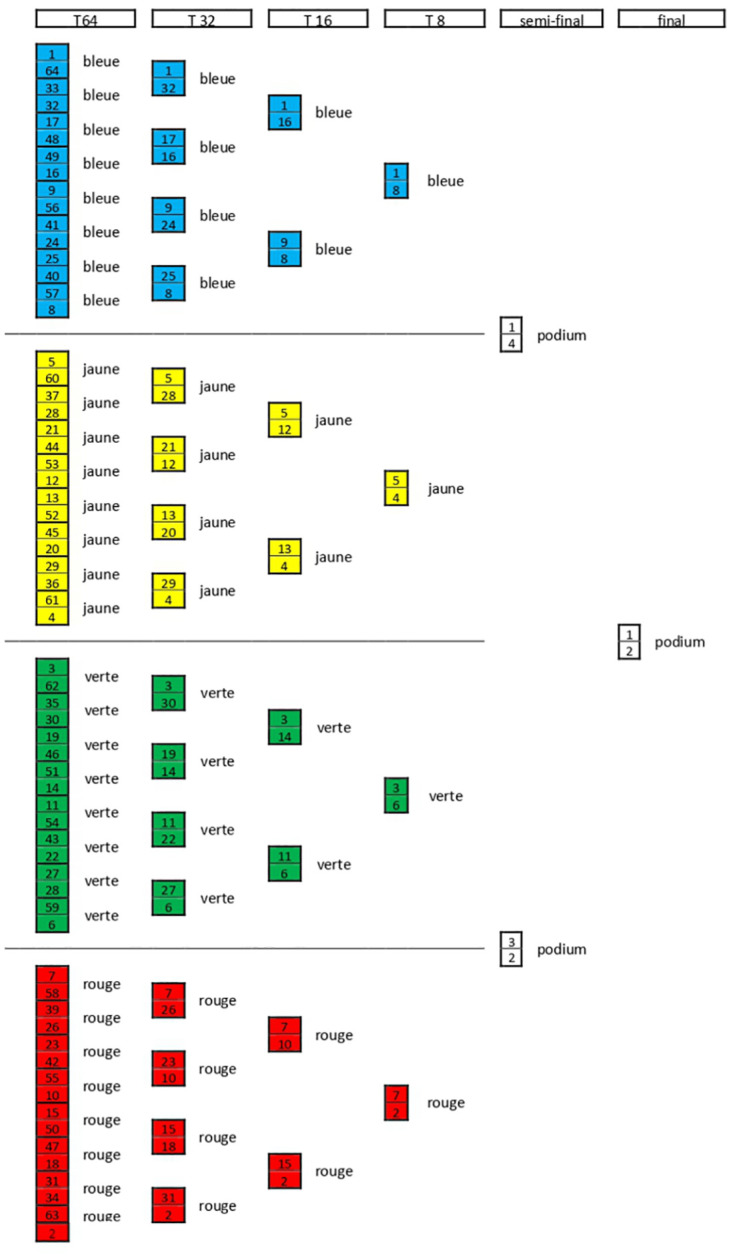
Sketch of the direct elimination table of a typical competition round, from 64 up to the final, scaling with decreasing powers of 2. Different colours identify different pistes. For clarity of the picture, larger tables (128, 256) have been omitted.

Usually, a competition (tournament) takes place over a single day and consists of two main phases, one round of eliminating pools (Table 1) followed by a direct elimination table (Fig 1).

**Table 1:** Allocation method explained for three different pools (A, B and C), based on ranking placements of 18 competitors. Each column represents a complete pool with 6 fencers.

Pool		A		B		C	
Fencer ranked		1	→	2	→	3	↓
	↓	6	←	5	←	4	←
	→	7	→	8	→	9	↓
	↓	12	←	11	←	10	←
	→	13	→	14	→	15	↓
	↓	18	←	17	←	16	←

The pools comprise 6 or 7 fencers, depending on the number of participants. They are composed taking into account the latest official FIE ranking, which collects the points obtained in the previous events of the current season or in the previous season on the basis of athletes’ placements.

The allocation of fencers in the pools follows the method shown in Table 1.

In pool rounds, each competitor fences a bout against all of the other members of their pool, up to five hits in only one period of three minutes and an extra priority minute in case of tie.

After the pools, a single general ranking of all the athletes is established, on the basis of the following indices: first, $\frac{V}{M}$ is considered, where *V* is the number of victories and *M* the number of bouts; then, in case of equality, the difference *HS* − *HR* between the hits scored (*HS*) and the hits received (*HR*) is taken into account; finally, in case of further equality in both $\frac{V}{M}$ and *HS* − *HR*, the fencer who has scored most hits (highest *HS*) is seeded highest. In the special case of absolute equality, the order is decided by drawing lots.

From the round of pools, only 70% of the fencers is qualified for the direct elimination phase, depending on the classification after pools.

Direct elimination table consists of many rounds that scale with decreasing powers of 2 (usually 256, 128, 64, 32, 16, 8, 4 and final) as shown in Fig 1: as we can see, the first classified after pools is coupled with number 64, the second one with number 63 one and so on.

For example, if the competition starts with 100 participants, after pools there are 70 athletes qualified to the direct elimination round, which is an incomplete table of 128 (since they are more than 64). Therefore, the last 12 athletes, from number 59 up to 70 after pools, have to win one more match to access the table of 64, while the first 58 fencers automatically advance. In detail, the couples are: 59–70, 60–69, 61–68, 62–67, 63–66, 64–65. Once these matches are completed, table of 64 starts for all the participants left, following the bouts indicated in Fig 1.

Intuitively, in each round the fencer who wins his/her bout have access to the next one, while the loser ends his/her competition and obtains a placement coincident with the reached round, according to the ranking after pools. For example, if fencer A loses against fencer B in the table of 16, fencer A can be placed in the classification among the 9^th^ and the 1^th^ place.

The general classification is compiled from the winner of the final bout, who is also the winner of the competition, followed by the second, the one who loses the bout for the first place; then, there is an *ex aequo* for the third place, assigned to the two fencers defeated at the round of semi-finals; the other placements are given as explained above.

The goal of attending competitions is to rise in the official FIE ranking. In fact, at the end of each tournament, all the participants gain a certain amount of points, fixed by the scale of Table 2.

**Table 2:** Scale points in the official FIE ranking.

1^st^ place	32 points
2^nd^ place	26 points
3^rd^ place *ex aequo*	20 points
5^th^–8^th^	14 points
9^th^–16^th^	8 points
17^th^–32^nd^	4 points
33^rd^–64^th^	2 points
65^th^–96^th^	1 point*
97^th^–128^th^	0.5 point**
129^th^–256^th^	0.25 point**
beyond 256^th^	0.1 point**

We can see that the scale decreases in a non-linear way, following a power law from the 9^th^ place on. Notice that some values were added later: * was introduced in season 2015/2016; ** were introduced only in 2019.

Ranking is not cumulative over years, instead it rolls during the season: the new result cancels out the previous year result in the corresponding competition. Moreover, the official Junior ranking of the FIE considers only the best six results of the World Cup events in which the fencer has participated, other than the Zonal and World Championships, for a total of eight results collected. As a consequence, only those athletes who actually attended at least one event in a season are listed in the ranking.

We need to specify that different kinds of competitions weight differently in ranking: points obtained in World Cups events are multiplied by a factor of 1; Zonal Championship points in our dataset are multiplied by a factor of 1.5 (this rule has been updated in season 2019/2020, the factor being reduced to 1); points obtained in World Championships are multiplied by a factor of 2.5.

### Model

In this section we provide an agent-based model realized in NetLogo environment [30] in order to reproduce, through numerical simulations, the dynamics of several international competitive seasons in fencing.

In section Fencing rules we introduced the main notions of the chosen fencing discipline (épée), whose combat features make it particularly suitable for our main goal, that is the evaluation of the relative role of talent and chance in determining successful careers of athletes (fencers) belonging to a certain community.

To this aim, in every simulation run we consider a given number *N*_*S*_ of seasons/years, each made of a certain number *N*_*T*_ of tournaments (also called events or competitions). At the beginning of each run, all the agents are randomly listed in an initial ranking. Then, every season, each athlete of the community can “choose” the number of events (≤ *N*_*T*_) he or she wants to participate during that year, with a probability related to the ranking order updated at the end of the previous season. Thus, each tournament is characterized by a different number *N* of participants (see S1 Appendix for more details).

Let us now describe how we model each single tournament, following the rules explained in the previous subsection.

#### Round of pools

For sake of simplicity, in our model every pool is built with 6 agents, on the basis of the scheme reported in Table 1. Thus, there will be a total of *N*/6 pools (*N* should be a multiple of 6). Inside a certain pool, a given competitor fences, in turn, with each one of all the other 5 competitors, keeping track of the victories, of the hits scored and of the hits received.

The sequence of touches occurring during a single match is realized by randomly choosing subsequent time intervals between 2 and 60 seconds (the upper limit is related to a specific épée rule, the lower considers a delay between the start commanded by the referee and the actual beginning of the two opponents’ actions). At the end of each interval, both athletes have the possibility to perform a valid touch according to the following quantity:

$$\begin{array}{c} P_{k}=a\;T_{k}+(1-a)L_{k},\;\;\;\;\;\;k=1,2 \end{array}$$ (1)

For each fencer, *P*_*k*_ depends both on his/her talent *T*_*k*_ and on the chance parameter *L*_*k*_. In analogy with previous studies [2, 3, 5, 6], we represent talent with a real variable *T*_*k*_ ∈ (0, 1], randomly extracted from a Gaussian distribution with mean *μ* = 0.6 and standard deviation *σ* = 0.1; thus, *T*_*k*_ envelopes all the inner qualities of an athlete (intelligence, skills, ability, training, motivation, etc.). Talent should be broadly interpreted as the maximum potential that can be expressed by the athletes throughout the competitions. Therefore, it is an intrinsic feature of each agent that we assume constant during an entire simulation run (made of several seasons). On the other hand, the chance parameter *L*_*k*_ is randomly extracted for each single touch in the interval $\left[{\bar{L}}_{k}-0.3,{\bar{L}}_{k}+0.3\right]$. This choice takes into account two different sources of randomness, acting on different temporal scales. The mean chance parameter ${\bar{L}}_{k}$ affects the average performance of the corresponding athlete during a tournament, due to external unpredictable factors that may influence that performance in the day of the competition (the organization of tournaments itself could be responsible of this, as mentioned in the Introduction). On that account, ${\bar{L}}_{k}$ it is randomly extracted in the interval [0.3, 0.7] at the beginning of each competition and remain fixed for the entire tournament. Of course, during each single match, other unpredictable factors can influence athlete’s performance on a shorter time scale, thus we let the chance parameter *L*_*k*_ to randomly fluctuate around its mean value ${\bar{L}}_{k}$ for every touch. For example, a fit agent could have ${\bar{L}}_{k}∼0.7$ and a chance parameter extracted from a uniform distribution in the interval [0.4, 1], clearly in his/her favour; on the other hand, an out of condition fencer could have ${\bar{L}}_{k}∼0.3$ with a consequent interval for *L*_*k*_ limited to [0, 0.6] while performing a valid touch. The common parameter *a* ∈ [0, 1] in Eq 1 touch represents the so called *talent strength*, i.e. the weight of talent in making the hit; as a consequence, (1 − *a*) weights the importance of chance. If *P*_1_ > *P*_2_ we assign a valid touch to the first fencer and his/her score is increased of 1; if *P*_2_ > *P*_1_ the opposite happens.

It is worth noting that the talent strength *a* is the only free global parameter in our model, which allow us to estimate—through the comparison with real data—the relative importance of talent and chance in the fencing discipline.

In a real fencing match, there is also the possibility to have one or more double-hits. This has been implemented in the model by allowing a given fraction *F*_*d*_ of touches to be considered a double-hit. On the basis of our experience (one of the authors—C.Z.—is also a fencer and fencing instructor), *F*_*d*_ can be defined as:

$$\begin{array}{c} F_{d}=0.4[\frac{1}{2}(1-\langle T\rangle )+\frac{1}{2}(1-\frac{\tilde{r}}{N})] \end{array}$$ (2)

and it is the result of two different contributions of equal weight: (1 − 〈*T*〉), where 〈*T*〉 represents the mean talent of the two opponents; $\left(1-\frac{\tilde{r}}{N}\right)$, being $\tilde{r}=\left|r_{1}-r_{2}\right|$ the difference between the initial ranking of the fencers considered. Thus, the possibility of a double-hit increases when both mean talent and ranking difference decrease. Notice that *F*_*d*_ is confined in the interval [0, 0.4] thanks to the prefactor in Eq 1 double: in correspondence of each new touch, a random variable *h* is extracted in the interval [0, 1] and, if *F*_*d*_ > *h*, a double-hit occurs. In this case both the competitors increase their scores of 1.

In case of a tie, “priority” takes place, as explained in Fencing rules. In the model, it is implemented as follows: with a coin flip, priority is assigned to one of the two opponents; then, the extra minute starts and only a single touch is allowed; if a double-hit occurs the score is not updated; if the minute ends without any single valid hit, whoever owns priority wins the bout. Notice that the coin flip for the priority assignment is a third source of randomness, acting on an intermediate time scale, independent of the first two and intrinsic to the fencing rules.

When all the pools are completed, a summary classification is established on the basis of several indices described in Fencing rules; as a consequence, the first 70% of the athletes after the round of pools can access to the direct elimination table.

#### Direct elimination table

Direct elimination table is built according to the classification after pools and can be complete or incomplete: in the former case, the number of competitors is an exact power of 2 and all bouts of that round must be held; in the latter, the number of athletes, equal to the vacancies in the table, can advance without facing any opponent.

**Fig 2:**
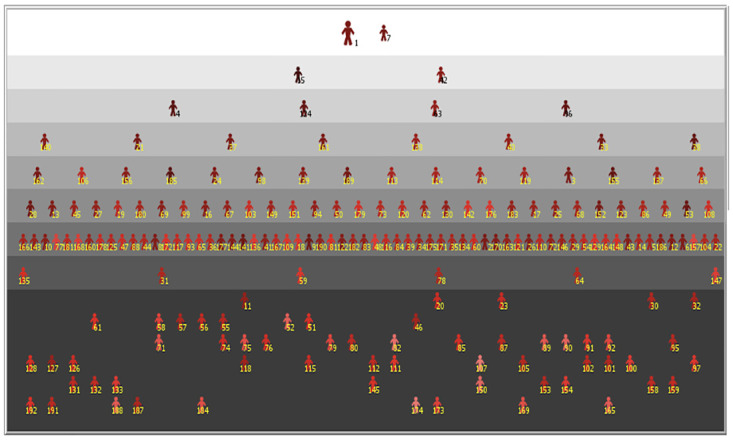
An example of athletes’ classification at the end of a simulated tournament. Subscripts display the initial ranking of the fencers, while their pyramidal arrangement indicates their final placements. Agents in the bottom, dark-gray, part of the figure are the 30% of athletes who did not access to the direct elimination table.

During this phase, the bout has its canonical structure, three periods of three minutes each and a maximum score of 15 points, each touch being assigned with the same procedure implemented for the bouts in the Round of pools (Eqs (1) and (2). Again, when the score is tied, an extra minute of priority is given, as described in Fencing rules and implemented in the same way as in Round of pools. The fencer who wins the bout advances in the table, while the loser ends his/her competition. This selective mechanism is the same in every round of the table and, at the end of the tournament, produces a pyramidal arrangement similar to that one shown in Fig 2: at the top level we found the first and the second classified; at the bottom level, the 30% of athletes who did not pass the round of pools; all the other fencers lie in the middle levels. Each agent is labelled with his/her position in the initial ranking: in the example considered, the winner of the competition started from the first position in the ranking, while the second classified started from the 7^th^ position, and so on.

At the end of the tournament, fencers receive an amount of points according to their classification, following Table 2 of Fencing rules. Notice that we do not distinguish between Championships and World Cups in our model, thus all events weight equally in simulation rankings. As already explained in Fencing rules, ranking is not cumulative over the years. On the contrary, each new result cancels out the result obtained in the corresponding competition of the previous year. The simulation stops when the last tournament of the last year ends. At this point it is possible to look at several output parameters, such as the final ranking of the athletes, the relationship between their initial and final placement (calculated either for each season or for each single competition) and even the interplay between talent and rank, all of them as a function of the selected value of talent strength *a*.

## Results and discussion

In the previous section we briefly showed the necessary features for simulating the careers of young fencers and how we inserted them in the structure of our model. Our aim is that of investigating fencing dynamics for different values of the global talent strength, comparing simulation results with data in order to evaluate the relative role of talent and chance in this sporting discipline.

As mentioned in section Dataset of Materials and methods, for this comparison we focus on épée rankings of Junior Men and Women. We set to *N*_*S*_ = 7 the number of seasons/years to simulate, adding an extra one (the first) as a trial stage for the following six (i.e. the longest possible career of young fencers). According to the average length of the official FIE rankings (Dataset), we consider a community of *N*_*M*_ = 600 fencers to simulate the Junior Men seasons and a smaller community of *N*_*F*_ = 500 fencers for the Junior Women ones.

For every season, we simulate *N*_*T*_ = 8 distinct tournaments with a variable number *N* of participants (see Fig 9 in S1 Appendix), following effective data results considered in official ranking. In fact, during a given season, each athlete of the community can “choose” the number of events (≤ *N*_*T*_) he or she wants to attend, with a probability related to the ranking order for that year. Those conditional probabilities have been extracted from the real dataset for both men and women, as explained in S1 Appendix (see Fig 10), and the model has been calibrated accordingly.

In Table 3 we summarise the setup of the fixed parameters that we adopt in our simulations, included mean and standard deviation of the Gaussian distribution of athletes’ talent introduced in the previous section. In order to have statistically significant results, we always average the outputs over 10 simulation runs, each starting from a different realization of the talent distribution among agents. There is no need to add runs since we already observe quite stable results and lower errors than those found in the data.

**Table 3:** The fixed set of parameters for each simulation run.

Parameters	Simulations	
	Men	Women
N-Years-to-simulate	7	7
N-Tournaments-per-year	8	8
Talent-gaussian-mean	0.6	0.6
Talent-standard-deviation	0.1	0.1
Total-athletes	600	500

**Fig 3:**
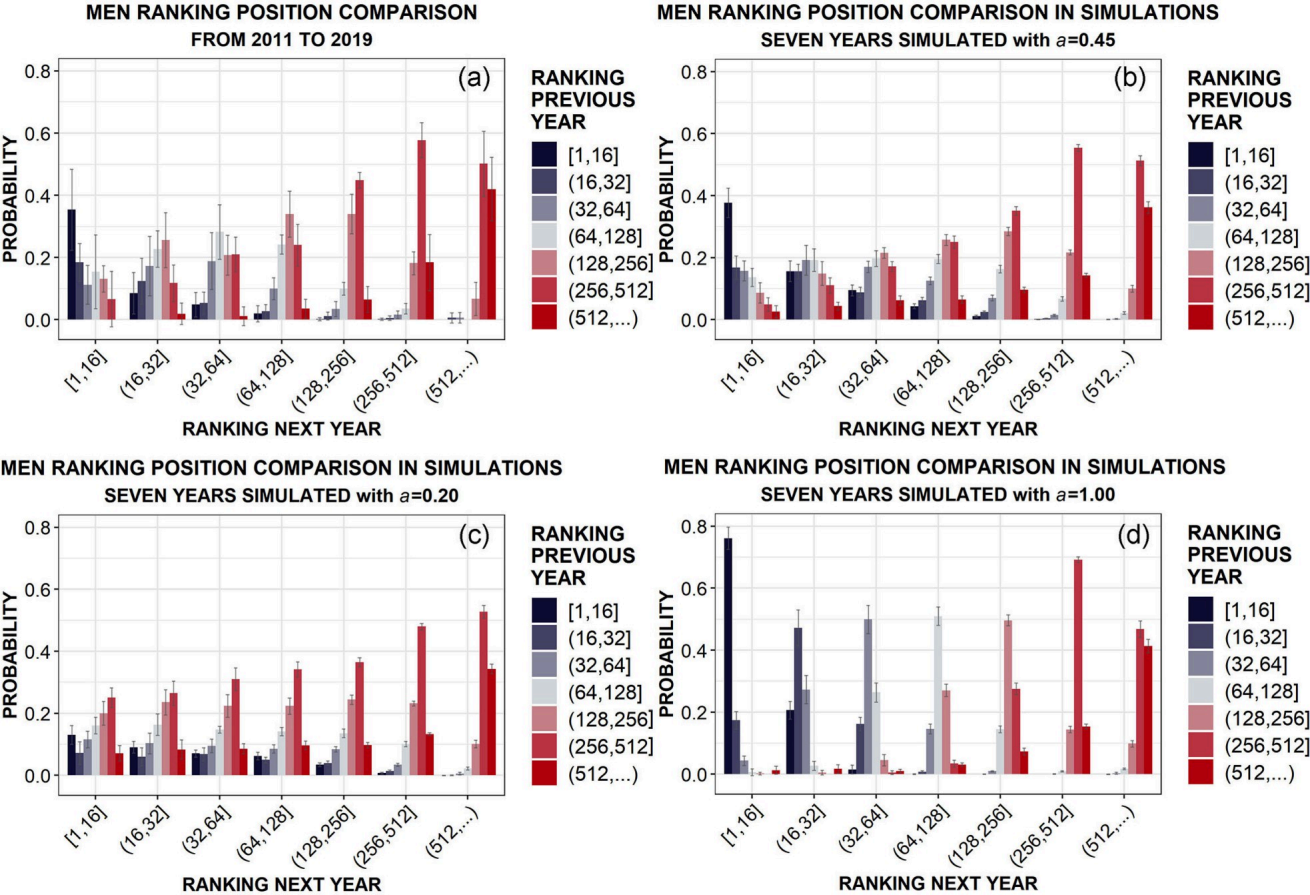
Comparison between data and simulations (averaged over 10 runs). Probability of having the same or a different placement the following year in Junior Men, given the associated ranking placement in the previous year. Ranking positions are arranged in groups of different sizes, to enhance visualization. Sketch of the panels: (a) data; (b) simulation with the optimal value of *a*; (c) simulation with a very low value of *a*; (d) simulation with the highest possible value of *a*. For each mean outcome, the corresponding standard deviation is also reported as an error bar.

**Fig 4:**
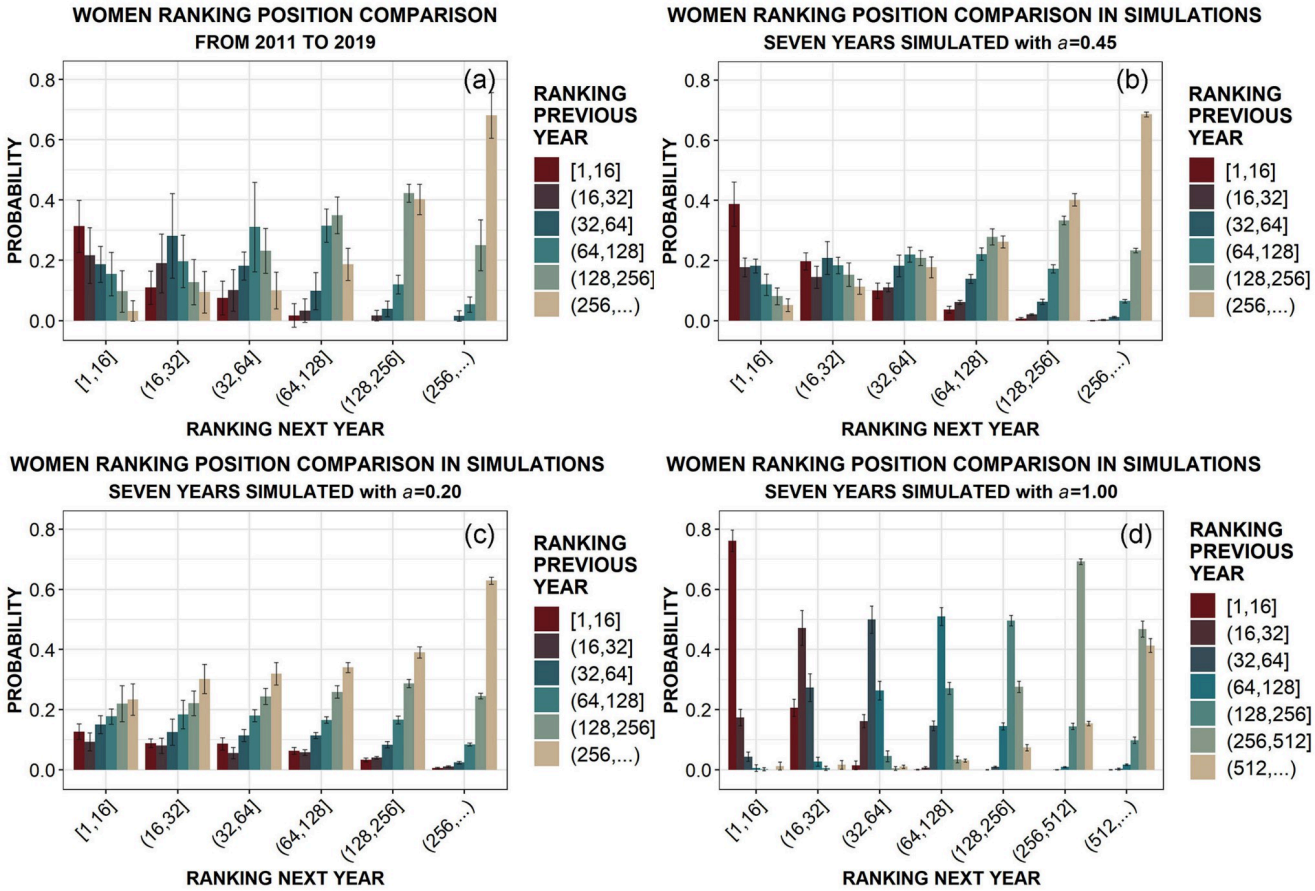
Comparison between data and simulations (averaged over 10 runs). Probability of having the same or a different placement the following year in Junior Women, given the associated ranking placement in the previous year. Ranking positions are organized in groups of different sizes, to enhance visualization. Sketch of the panels: (a) data; (b) simulation with the optimal value of *a*; (c) simulation with a very low value of *a*; (d) simulation with the highest possible value of *a*. For each mean outcome, the corresponding standard deviation is also reported as an error bar.

Our goal is to find the optimal value of the talent strength *a* able to produce the best agreement between data and simulations. To do so, we first consider the probability of improving, maintaining or worsening the ranking placement obtained the previous year. In fact, if the official ranking was a perfect mirror of athletes’ talent, those probabilities should be peaked in the corresponding placements, season after season, with very small fluctuations (Figs 3 and 4). Instead, observing the results of real data analysis in panels (a) of both Fig 3, for men, and Fig 4, for women, there is only a weak correlation between previous and following ranking positions: for example, athletes who conclude a certain season in the first 16 positions in the ranking, at the beginning of that season were in the same first 16 positions only with a probability between 0.3 and 0.4, while are slightly less likely to have started from lower positions in the ranking, and have still a not negligible probability to have started below the 500^th^ position. This effect is even more pronounced for the other placements, suggesting that talent explains only half of the story: evidently, the influence of external factors cannot be neglected and a certain role of chance should be also taken into account.

In order to quantitatively estimate that role, we report in the other panels of Figs 3 and 4 the analogous results obtained with our simulations for different values of the talent strength *a*. The simulation outputs yielding the best agreement (inside the error bars) with data correspond to *a* = 0.45, as shown in panels (b) of both figures. On the other hand, the results for smaller (*a* = 0.2) or greater (*a* = 1) values of the talent strength are clearly not compatible with data, as reported in panels (c) and (d) of both the figures. The selection of the optimal value *a* = 0.45 is supported by the mean squared error estimation, as shown in detail in S1 Appendix (see Fig 12, top panels). Those first findings suggest that the role of chance in épée competitions, estimated by the factor (1 − *a*) in Eq (1) touch, is absolutely not negligible if compared with talent: actually, it seems to be quite consistent, even slightly above 50% for both Junior Men and Women.

**Fig 5:**
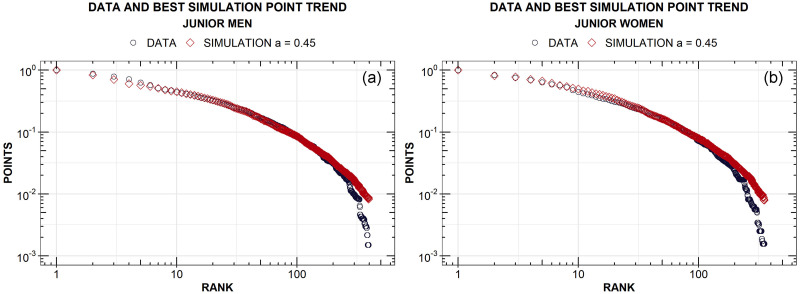
Trend of the average total points in Junior rankings, normalised to their maximum value, compared to simulations for male (a) and female (b) fencers.

To further support the choice of *a* = 0.45 as the best candidate for talent strength, we can move forward with other comparisons between data and simulations. In particular, it is interesting to look at the normalised points cumulated by real athletes at the end of the season, as a function of their final ranking, and compare them with the analogous ones obtained through simulations if we change *a*. A very good agreement emerges for values included in a narrow range between *a* = 0.4 and *a* = 0.6 but, again, the lowest mean square error is obtained for *a* = 0.45 (see the bottom panels of Fig 12, in S1 Appendix). The corresponding curves, obtained averaging over all the considered seasons and normalized to their maximum value, are reported in Fig 5. for both men (a) and women (b). One can notice significant overlaps, which are almost indistinguishable for the first 200 placements. For ranking positions higher than 200, data and simulation points act differently: the former decrease more rapidly, which can be due to a finite size effect more pronounced in data since ranking lengths vary over time, other than to an update of the rules for the point scale, as mentioned in section Fencing rules.

Summarising our first results of the study of fencing competitions along several seasons, we should conclude that: (i) data analysis already shows an evident role of external factors (chance) in determining the athletes’ placements at the end of each season; (ii) the comparison with the simulation outputs of our fencing model allow us to quantify this role by tuning the talent strength parameter and finding the value which minimizes the error. That optimal value is *a* = 0.45, the same for both men and women, so we fix it to explore single tournaments through our model.

Specifically, we observe the relationship between initial ranking and final placement at the end of a given competition. To do so, we average over 80 events for both male and female athletes in simulations, and over 52 and 48 events respectively for men and women in our dataset (see S1 Appendix). We monitor the top sixteen fencers in the ranking or in the final classification. For those athletes, we would like to ask the following two questions:

1. What is the conditional probability of obtaining a certain final placement in the tournament provided that one starts from a certain initial ranking position?
2. What is the conditional probability of having started from a certain position provided that one reaches a certain final placement in the tournament?

**Fig 6:**
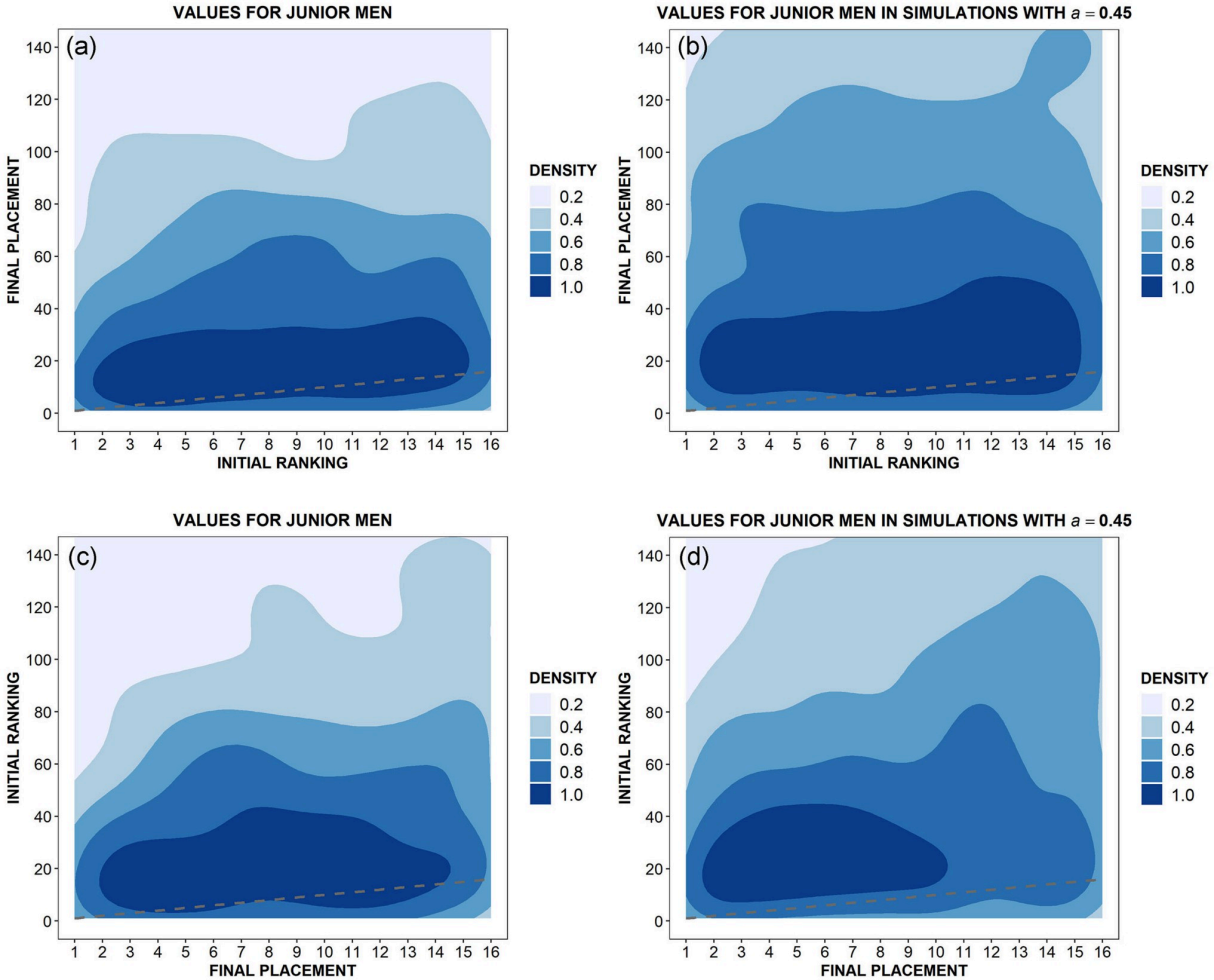
Single tournaments: Kernel density plots for initial ranking position versus final classification (top panels) and vice-versa (bottom panels) in both World Cups (a-c) and simulations (b-d) for male fencers. In every panel, a dashed gray line shows the ideal case in which talent would be the only variable influencing both ranking placements and tournaments’ results.

**Fig 7:**
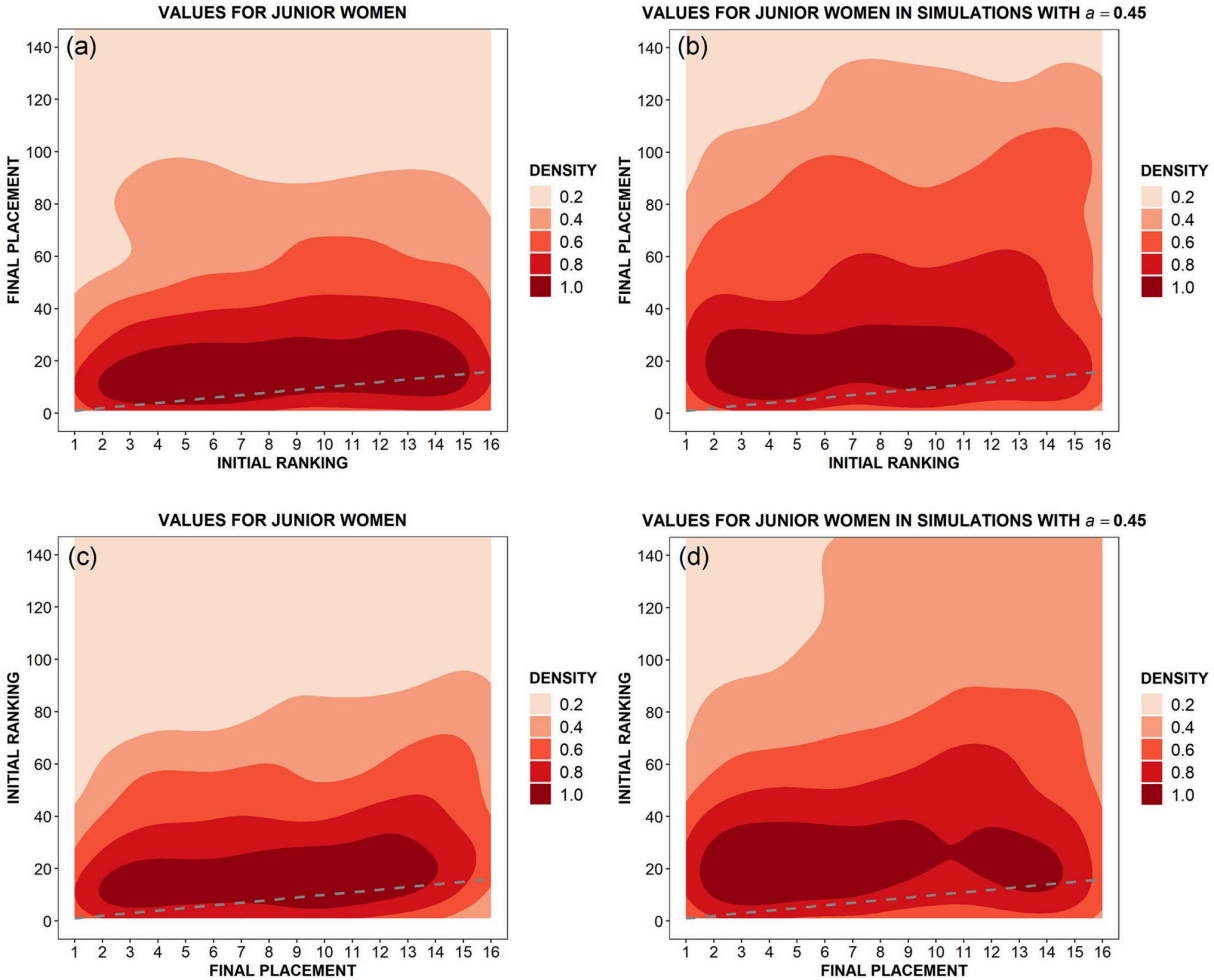
Single tournaments: Kernel density plots for initial ranking position versus final classification (top panels) and vice-versa (bottom panels) in both World Cups (a-c) and simulations (b-d) for female fencers. In every panel, a dashed gray line shows the ideal case in which talent would be the only variable influencing both ranking positions and tournaments’ results.

In the ideal case in which talent would be the only variable influencing both ranking placements and tournaments’ results, these two variables should be strongly correlated and such a correlation should be highlighted by a suitable density kernel analysis [31]. Thus, since we already found that the role of chance in épée competitions matters at least as much as that of talent, one should expect important deviations from this ideal behaviour. Moreover, one could also expect that our model should be able to numerically reproduce those deviations. That is precisely what we observe in Fig 6, for male fencers, and in Fig 7, for female ones, where the (normalised) density kernel plots are reported both for data, panels (a)—(c), and for simulation, panels (b)—(d). Looking at those figures one can draw the following conclusions:

1. In all the panels, the observed behaviour is very far from the ideal case, represented by a dashed gray line.
2. The comparison between panels (a) and (b) shows that our model captures the fact that the first 16 (male and female) athletes in the ranking have a quite high probability to reach a final placement included in approximately the first 30 positions, but—evidently due to the consistent role of chance—have also a decreasing, not negligible, probability to finish the tournament in lower (and sometimes much lower) positions.
3. At the same time, regarding panels (c) and (d), simulation results essentially reproduce the analogous effect observed in data, where (male and female) athletes placed in the first 16 positions at the end of a tournament came from the first 30 or 40 positions of the initial ranking, but with a not negligible decreasing probability to come also from lower positions.

One last consideration about talent. In general, from the analysis of data about sport disciplines it is not possible to directly extract the distribution of talent, being the latter an hidden variable of the individuals. However, one could think that a strong correlation between ranking and talent should be, in some way, preserved by the competitive selection of tournaments. Thus, athletes in the top ranking positions are usually assumed, by definition, to be the most talented and, vice-versa, those in the bottom positions the less gifted. Unfortunately, the latter is exactly the kind of assumption which should be questioned in disciplines where success is noticeably influenced by chance, as we have shown for épée fencing. Therefore, we can intuitively expect some violations of this assumption in fencing competitions.

**Fig 8:**
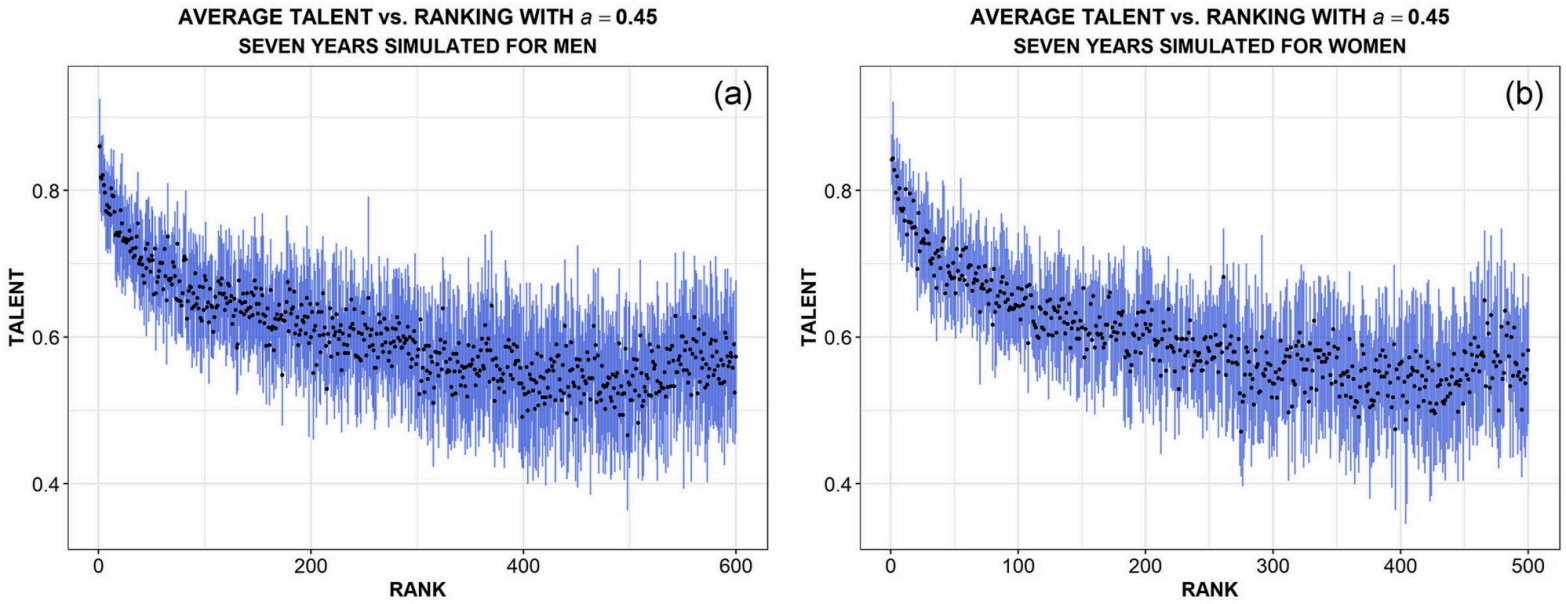
Distribution of talent as a function of fencers’ ranking in simulations, for men and women respectively panel (a) and (b). Mean values from the seventh year of ten runs are shown, error bars (blue) representing their standard deviation.

Simulations help us to confirm such an intuition, since we assign a fixed talent to all the agents at the beginning of a given simulation run, thus we are able to report their talent as a function of their final ranking. This has been done in Fig 8 for men (a) and women (b), respectively. Black points indicate the mean talent of athletes for each position in the ranking at the end of seven seasons/years, averaged over 10 simulation runs. Error bars are the corresponding standard deviations. In both panels we observe an initial rapid decreasing trend of talent, but with strong fluctuations which progressively increase as ranking gets worse. Such a behaviour implies that, if it is true that, on average, no athletes with talent below the mean (0.6) can be found in the best 50 positions, it is also true that very talented athletes (one standard deviation above the mean) can be detected at any position in the ranking. Moreover, the trend starts to slowly bend upward approaching the last positions, an effect that likely takes into account the possibility that certain pretty talented agents could attend very few competitions during the chosen interval of time. In the real world, they may represent new fencers at the beginning of their career, perhaps the youngest ones, who start competing without any ranking points even if they are as talented as other older participants.

## Conclusions

In this paper, we investigated the role of talent and chance in individual sports, studying fencing, a combat sport based on direct elimination tournaments. We collected [13, 22–28] and analysed data on international rankings and on World Cup results of male and female épée fencers under 20 years old. Then, we built an agent-based model, calibrated on those data, which allowed us to estimate the relative weight of chance (external factors, random lucky or unlucky events) with respect to talent in fencing competitions. We find that this weight is quite high, around 50% for both men and women. Following the line of reasoning expressed in the conclusive remarks of Ref. [3], we could claim that the peculiar rules of épée make this fencing discipline the best candidate to provide the upper limits of chance contribution in sport competitions with individual scores, whereas the Olympic 100-meter dash studied in Ref. [3], with its 4% for men and 6% for women, would give the lower limits. Thus, in a hypothetical spectrum, these two disciplines would probably represent the extremes, with all the other individual sports—high jump, long jump, tennis, golf, car racing, motorcycle racing, etc.—in between. That can be verified using our model, which can be easily extended to other sports, especially to those which are tournament-based, like tennis. And if that were true, it would seriously question the methods of awarding cash prizes which, typically, follow an exponentially decreasing trend going from the first classified to the last one. Those methods are based on the implicit assumption of a one-to-one correspondence between talent of athletes and their performance in competitions. But we have shown that such a perfect correspondence probably does not exist, since in all these sports chance and randomness could heavily influence the performance of any athlete, thus the awarding rules should be revised in order to make them closer to reality.

## Supporting information

S1 Appendix Dataset and model insights [32–35]. (PDF)
