# Supplementary material for: On the role of chance in fencing tournaments: An agent-based approach
Source: PLoS One. 2022 May 5;17(5):e0267541. doi: 10.1371/journal.pone.0267541 (PMC9070931; doi:10.1371/journal.pone.0267541)
Supplement: S1 Appendix — (PDF) [file pone.0267541.s001.pdf]

S1 Appendix. Dataset and model insights. In the first two sections of Materials and methods we presented our dataset and introduced the main rules of fencing, with particular regard to épée discipline, as well as the competition formula adopted by the type of events which we are interested in. Other than rankings from 2011 to 2019 (see Dataset), our data comprise 52 results for Junior Men and 48 for Junior Women, considering only events with at least 100 competitors. We observed that the number of participants in a competition can reach a maximum value of 280 for Men and 230 for Women, with an average around 170 and 150 respectively, as visible in Fig. 9.

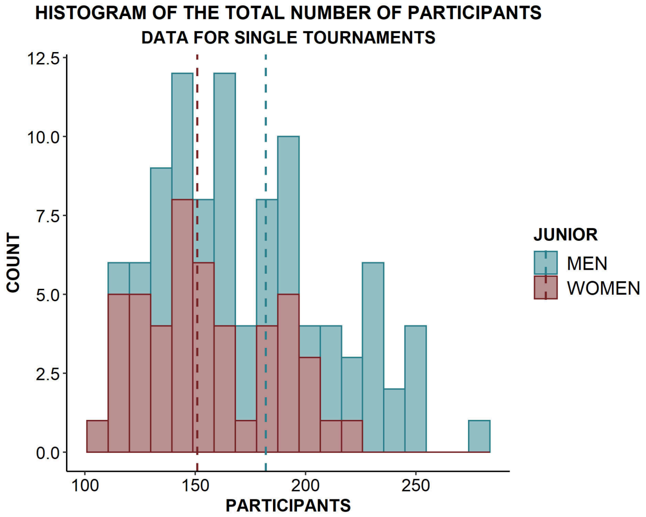

Fig 9. Histograms showing the total number of participants in every event, i.e. Junior Men and Women World Cups, from 2008 to 2020.

From the variety of participation in tournaments, it follows that the list of participants in single competitions cannot have a fixed length either. That is why we let the number of participants N vary in simulation runs (see Results and Discussion). It is quite natural that athletes attend only a certain number of events during a given season, according to their position in the ranking and to their country's regulations. One could think that the higher the position in ranking, the more events the athlete took part in. Data analysis for both men and women shows that this is generically correct, but we find a non-trivial probability distribution of ranking placements conditional to the actual attended events (see Fig. 10) which allowed us to calibrate the model.

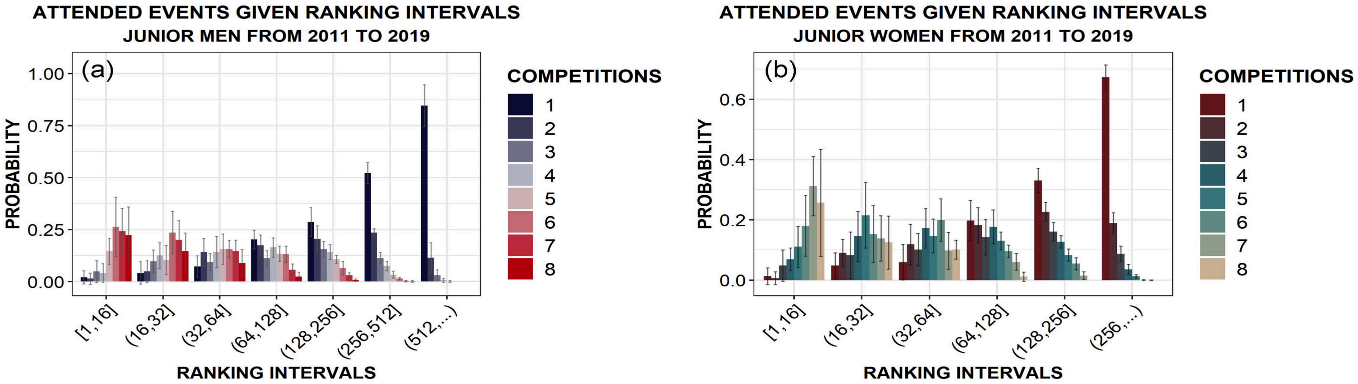

Fig 10. Probability of having attended a certain number of Junior competitions in data, given the associated ranking placement in the same year. Mean values with their standard deviation for male fencers are shown in the left panel (a), the analogous for female in the right panel (b).

If we focus on the first sixteen athletes, who are usually considered the top performers, we can examine the relationship between ranking positions before a certain competition and the final results of that same event, and vice-versa, for both men and women. We evaluate the median value of the initial/final ranking for the top sixteen fencers, rather than the mean value, because of the asymmetric nature of data themselves. In Fig. 11 we show the results. The shadows indicate the median absolute deviation, a measure of spread suitable for median values.

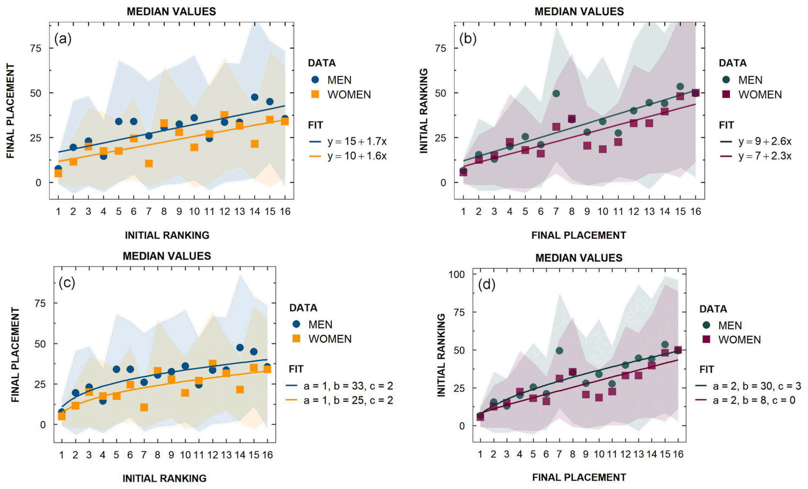

Fig 11. Median values for the first sixteen Junior fencers, fitted in two different ways. In detail: panel (a) shows the relationship between initial ranking position and final classification in World Cups (from 2008 to 2020) with linear fits; panel (b) shows the relationship between final classification and initial ranking position in World Cups (from 2008 to 2020) with linear fits; panel (c) shows the relationship between initial ranking position and final classification in World Cups (from 2008 to 2020) with non-linear (quadratic) fits; panel (d) shows the relationship between final classification and initial ranking position in World Cups (from 2008 to 2020) with non-linear (quadratic) fits. Shadows correspond to the median absolute deviation, for a measure of spread.

In the upper panels, men and women data are fitted with linear functions, while in the lower panels are fitted with a non linear (quadratic) function. In Table 4 the fitting parameters for both functions are reported with the corresponding errors. They are quite high, reflecting the wide uncertainty of data.

Data	Linear Fit $y = q + mx$			Non Linear Fit $y = (ax^2 + bx)/(x + c)$			
	$q \pm \sigma_q$	$m \pm \sigma_m$	RSE	$a \pm \sigma_a$	$b \pm \sigma_b$	$c \pm \sigma_c$	RSE
Men IN-FIN	15 ± 3	1.7 ± 0.4	6.5	0.8 ± 1.1	33 ± 21	2 ± 2	6.2
Men FIN-IN	9 ± 4	2.6 ± 0.4	7.3	1.8 ± 1.6	30 ± 46	3 ± 7	7.3
Women IN-FIN	10 ± 3	1.6 ± 0.3	6.3	0.8 ± 1.2	25 ± 26	2 ± 5	6.3
Women FIN-IN	7 ± 3	2.3 ± 0.3	6.7	2.2 ± 0.6	8 ± 13	0.3 ± 2.4	6.9

Table 4. Parameters and residual standard error (RSE) of both linear and non linear fits, which are calculated on the median values of initial versus final rankings in single tournaments and vice-versa. The abbreviation 'IN-FIN' stands for 'Initial ranking - Final placement' and relates to the axes in Fig. 11, while 'FIN-IN' means the opposite, 'Final placement - Initial ranking'.

Taking into account the information available from data, expressed by the fixed parameters of our model (see Table 3 in Model), we ran several simulations varying only talent strength a (Eq. (1)). For every value of a, we collected 10 simulation runs and we derived the mean squared error for the two main properties we compared with data: the probability of having the same or a different ranking position in two consecutive years; the trend of average total points as a function of ranking order (see Results and Discussion). Results are plotted in Fig. 12; they suggest that $a = 0.45$ is the optimal value for reproducing data, with an uncertainty of 0.5.

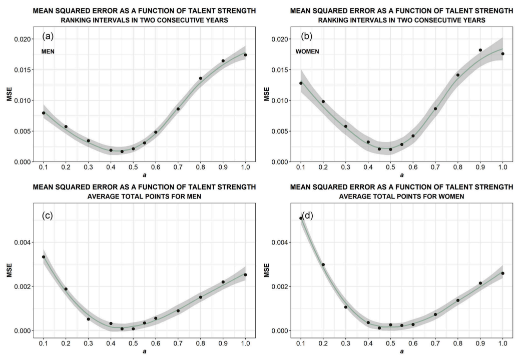

Fig 12. Evaluation of the mean squared error to find the optimal value of a. Panels (a) and (b) refer to the comparison between data and simulations of the probability of having the same or a different ranking position in two consecutive years; panels (c) and (d) relate to the trend of average total points as a function of ranking. Plots on the left show the values for men, those on the right for women.

We now focus on total points as a function of ranking order, highlighting their non-linear trend by testing four different fits with heavy-tailed behaviour: power law, log-normal, stretched exponential and power law with exponential cut-off (also known as truncated power law) curves. Following the formalism used in Ref. [32] (except for the log-normal function that here is shown in a more general form), we rely on the equations of Table 5, using a Levenberg-Marquardt algorithm [33] to perform the fits. As already mentioned in Results and Discussion, total points are averaged over the seasons and normalised to their maximum value. Results of the fits are summarised in Table 6 and plotted in Fig. 13. Examining data and simulation fits in Table 6, we found very similar fitting parameters, as supported by the comparison between panels (a)-(b) for Men and (c)-(d) for Women.

Function	Equation
Power law	$y \sim x^{-\alpha}$
Truncated power law	$y \sim x^{-\alpha} e^{-\lambda x}$
Stretched exponential	$y \sim x^{\beta-1} e^{-\lambda x^{\beta}}$
Log-normal	$y \sim \frac{1}{x} \exp\left[-\frac{1}{2}\left(\frac{\log x - b}{c}\right)^2\right]$

Table 5. Functional forms of the four different fits tested on average total points as a function of ranking order in both data and simulations.

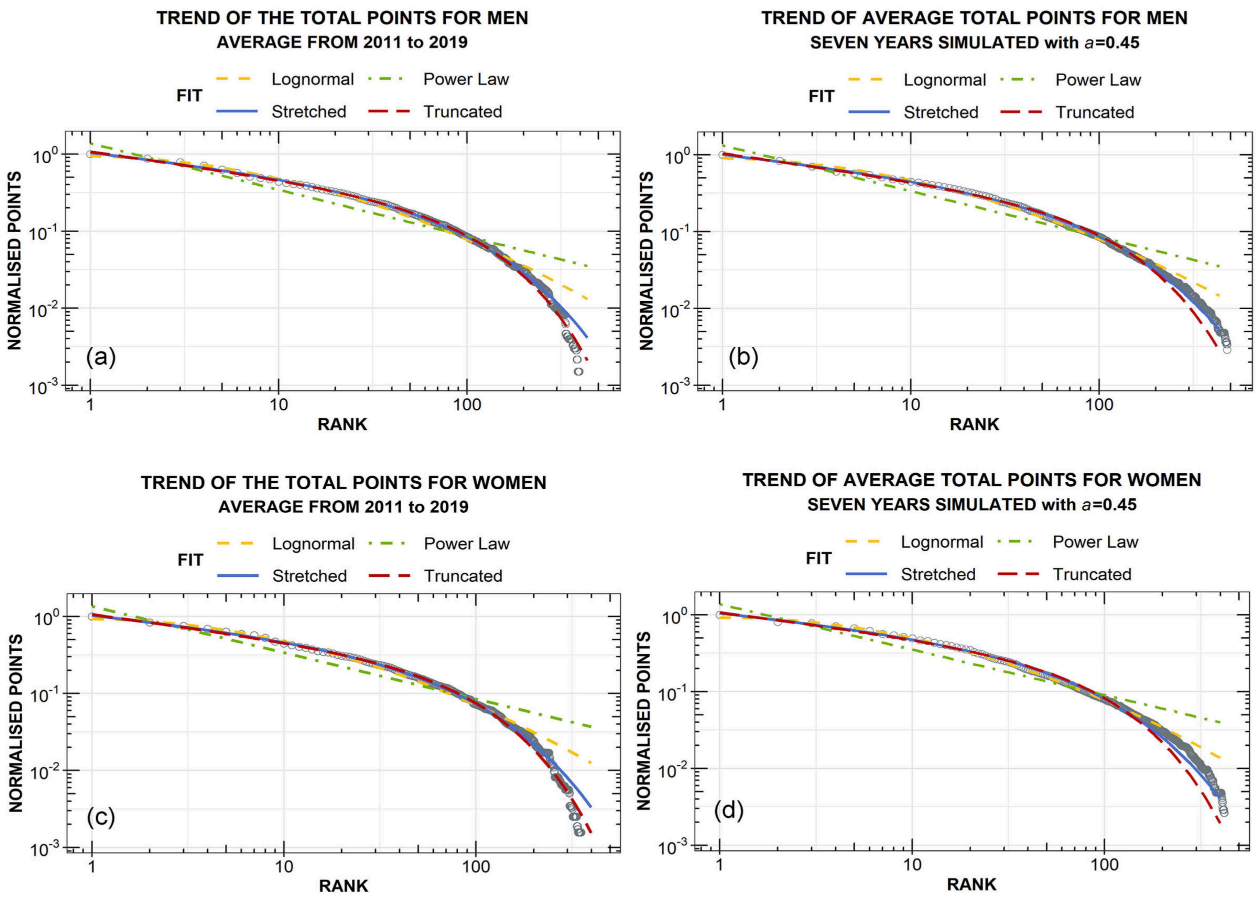

Fig 13. Comparison of the non-linear fits tested on both data (left panels) and simulations (right panels) for men ((a)-(b)) and women ((c)-(d)). The stretched exponential line (blue, solid) captures very well the trend of the normalised total points of Junior Men and Women (real and simulated). It is worth noting that the truncated power law has good agreement with data, less with simulation values, while log-normal curves are closer to simulation than to data points. In any case, power law fitting is the poorest, since it fails in reproducing the tail of those curves.

Preferring one fit to another is beyond our purposes, since we are mainly interested in highlighting a general "rich-get-richer" phenomenon in point accumulation as a function of ranking; nonetheless, we performed AIC model selection [34] for Women and Men data and simulations, observing that AIC is minimized by the stretched exponential function.

Finally, we checked that the conditional probabilities of having attended a certain number of competitions for the simulated fencers, given the associated ranking placement in the same year, are consistent with the data-driven ones, previously adopted for the calibration of the model (already shown in Fig. 10). Looking at Fig. 14, we can ascertain such a correspondence both for men (a) and women (b). Additionally, simulation results give back a variable number of participants to the tournaments during a given season that range from $N = 186$ to $N = 276$ for men and from $N = 156$ to $N = 216$ for women, both consistent with our dataset.

We specify that the statistical analysis was performed using R software [35].

Non-linear Fit	Total Points	Parameter $\pm$ Standard Error		Residual Standard Error
Power law		$\alpha \pm \sigma_\alpha$	/	
	Data Men	0.603 ± 0.008		0.042
	Simulation Men	0.596 ± 0.007		0.038
	Data Women	0.601 ± 0.009		0.045
	Simulation Women	0.591 ± 0.009		0.045
Truncated power law		$\alpha \pm \sigma_\alpha$	$\lambda \pm \sigma_\lambda$	
	Data Men	0.341 ± 0.002	0.0096 ± 0.0001	0.0063
	Simulation Men	0.345 ± 0.002	0.0088 ± 0.0001	0.0062
	Data Women	0.329 ± 0.003	0.0115 ± 0.0001	0.0069
	Simulation Women	0.320 ± 0.004	0.0111 ± 0.0002	0.011
Stretched exponential		$\beta \pm \sigma_\beta$	$\lambda \pm \sigma_\lambda$	
	Data Men	0.733 ± 0.002	0.0458 ± 0.0003	0.0053
	Simulation Men	0.727 ± 0.001	0.0438 ± 0.0002	0.0039
	Data Women	0.745 ± 0.002	0.0493 ± 0.0003	0.0052
	Simulation Women	0.749 ± 0.002	0.0463 ± 0.0004	0.0072
Log-normal		$b \pm \sigma_b$	$c \pm \sigma_c$	
	Data Men	4.41 ± 0.04	2.14 ± 0.02	0.013
	Simulation Men	4.60 ± 0.04	2.20 ± 0.02	0.012
	Data Women	4.20 ± 0.04	2.07 ± 0.02	0.013
	Simulation Women	4.25 ± 0.02	2.05 ± 0.01	0.0089

Table 6. Summary of the results for several non linear fits of ranking points in Junior category, both for data and simulations with the chosen talent strength $a = 0.45$.

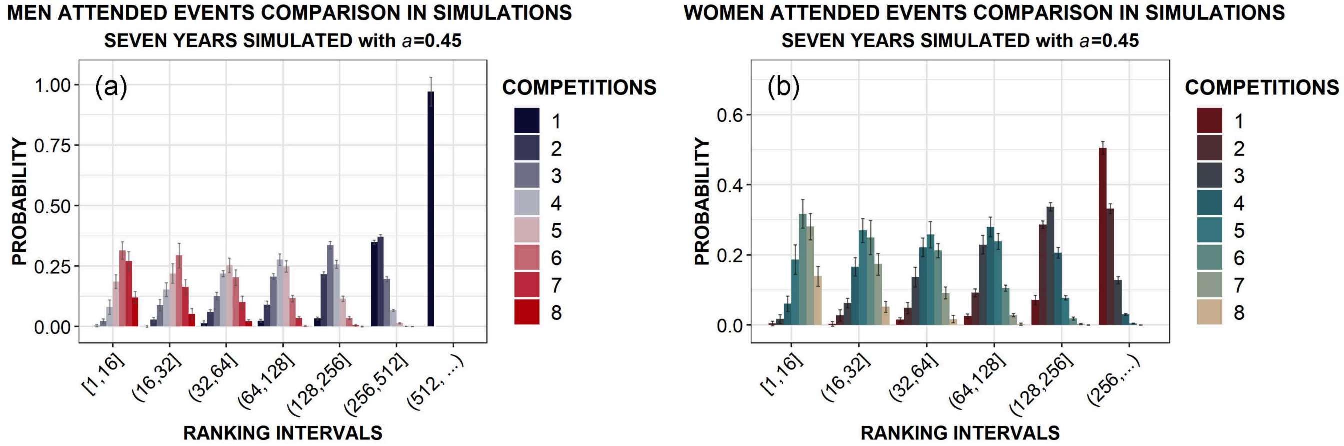

Fig 14. Simulations with $a = 0.45$: probability of having attended a certain number of competitions in Junior category, given the associated ranking placement in the same year. Mean values with their standard deviation for male fencers on the left, the analogous for female fencers on the right.
